# Type VI Secretion System Toxins Horizontally Shared between Marine Bacteria

**DOI:** 10.1371/journal.ppat.1005128

**Published:** 2015-08-25

**Authors:** Dor Salomon, John A. Klimko, David C. Trudgian, Lisa N. Kinch, Nick V. Grishin, Hamid Mirzaei, Kim Orth

**Affiliations:** 1 Department of Molecular Biology, University of Texas Southwestern Medical Center, Dallas, Texas, United States of America; 2 Department of Biochemistry, University of Texas Southwestern Medical Center, Dallas, Texas, United States of America; 3 Howard Hughes Medical Institute, University of Texas Southwestern Medical Center, Dallas, Texas, United States of America; 4 Department of Biophysics, University of Texas Southwestern Medical Center, Dallas, Texas, United States of America; University of Washington, UNITED STATES

## Abstract

The type VI secretion system (T6SS) is a widespread protein secretion apparatus used by Gram-negative bacteria to deliver toxic effector proteins into adjacent bacterial or host cells. Here, we uncovered a role in interbacterial competition for the two T6SSs encoded by the marine pathogen *Vibrio alginolyticus*. Using comparative proteomics and genetics, we identified their effector repertoires. In addition to the previously described effector V12G01_02265, we identified three new effectors secreted by T6SS1, indicating that the T6SS1 secretes at least four antibacterial effectors, of which three are members of the MIX-effector class. We also showed that the T6SS2 secretes at least three antibacterial effectors. Our findings revealed that many MIX-effectors belonging to clan V are “orphan” effectors that neighbor mobile elements and are shared between marine bacteria via horizontal gene transfer. We demonstrated that a MIX V-effector from *V*. *alginolyticus* is a functional T6SS effector when ectopically expressed in another *Vibrio* species. We propose that mobile MIX V-effectors serve as an environmental reservoir of T6SS effectors that are shared and used to diversify antibacterial toxin repertoires in marine bacteria, resulting in enhanced competitive fitness.

## Author Summary

The bacterial type VI secretion system (T6SS) is a contact-dependent protein secretion apparatus that is emerging as a major component of interbacterial competition in the environment. The bacterium *Vibrio alginolyticus* is a pathogen of marine animals and a causal agent of wound infections, otitis, and gastroenteritis in humans. In this study, we provide a comprehensive characterization of the environmental regulation, antibacterial activities, and secreted effector repertoires of the two T6SSs found in this pathogen. We also identify a subset of T6SS effectors that appear to be mobile and shared between marine bacteria that can interact with each other in aquatic environments. Our findings suggest that bacteria can incorporate T6SS effectors from competitors in the environment. These newly acquired toxins may be used to expand and diversify T6SS effector repertoires and enhance bacterial fitness.

## Introduction

The type VI secretion system (T6SS) is a protein secretion apparatus found in Gram-negative bacteria [1]. While it was originally described as a bacterial virulence determinant [2–4], subsequent findings demonstrated that many T6SSs are used as antibacterial determinants in interbacterial competition [5–8]. This tightly regulated macromolecular secretion apparatus functions similarly to a contractile phage tail but in a reverse orientation [1]. Upon perception of an extracellular signal, the secreted tail tube complex, composed of an inner tube made of stacked hexameric rings of Hcp that are capped by a trimer of VgrG and a PAAR repeat-containing protein, is propelled outside of the cell and into an adjacent recipient cell [1,9,10]. This tail tube is decorated with effector proteins containing toxic activities, either as domains fused to components of the tail tube or as proteins that bind to them [11]. Several T6SS effectors have been identified and found to cause toxicity through various mechanisms such as actin cross-linking [3], nuclease activity [12,13], and pore-forming [14]. In addition, two effector superfamilies with antibacterial peptidoglycan-hydrolase and phospholipase activities have been described [6,7]. Several proteins containing Rearrangement hotspot (Rhs) repeats were also suggested to be T6SS effectors [12,15].

We recently identified a widespread class of polymorphic T6SS effectors called MIX-effectors [16]. These effectors share an N-terminal motif named MIX (Marker for type sIX effectors) and have polymorphic C-terminal domains with diverse predicted antibacterial or anti-eukaryotic activities [16]. Notably, T6SS effectors that possess antibacterial activities are encoded in bicistronic units together with a gene that encodes for their cognate immunity protein that protects the cell against self-intoxication [6,7]. Up to six T6SSs can be encoded within a single bacterial genome [17], and each system can be differentially regulated [18–21].

*Vibrio alginolyticus*, a Gram-negative, halophilic marine pathogen associated with wound infections, otitis and gastroenteritis, is one of the most commonly reported disease-causing *Vibrio* species in the United States [22], and was also recently found to be a cause of coral diseases [23,24]. It encodes two T6SSs (VaT6SS1 and VaT6SS2) [25]. Sheng *et al*. previously reported several transcription factors and regulators that control the activation of *V*. *alginolyticus* T6SS1 (VaT6SS1) in the EPGS strain [25,26]. More recently, we found that VaT6SS1 of the *V*. *alginolyticus* 12G01 strain functions as an antibacterial determinant, and identified a MIX-effector, V12G01_02265 (hereafter we will use the prefix Va instead of the locus prefix V12G01_, thus the aforementioned protein is Va02265), that mediated antibacterial toxicity and is paired with an immunity protein, Va02260 [16].

In a previous study, we characterized the environmental conditions and cues that activate the two T6SSs found in the marine pathogen *V*. *parahaemolyticus* [20,27], and identified secreted effectors that mediate the antibacterial activity of the *V*. *parahaemolyticus* T6SS1 (VpT6SS1) [16]. However, we found no role for VpT6SS2 [20]. The two *V*. *alginolyticus* T6SS gene clusters, encoding VaT6SS1 and VaT6SS2 (S1A Fig), are similar to the *V*. *parahaemolyticus* T6SS clusters in both gene content and organization [20]. However, the environmental conditions that activate the *V*. *alginolyticus* T6SSs and whether they differ from the conditions that regulate the T6SSs in *V*. *parahaemolyticus*, the activity of VaT6SS2, and the *V*. *alginolyticus* T6SSs effector repertoires, remain unknown.

In this work, we set out to characterize the T6SSs in *V*. *alginolyticus*. We found that the *V*. *alginolyticus* T6SSs are differentially regulated by salinity and temperature, and that both systems can mediate bacterial killing during interbacterial competition. Using comparative proteomics, we identified several T6SS effectors, including MIX-effectors, that mediate antibacterial killing. Finally, we found a subset of mobile T6SS MIX-effectors that are shared between marine bacteria via horizontal gene transfer, and showed that such a mobile MIX-effector from *V*. *alginolyticus* can be transferred into *V*. *parahaemolyiticus* and retain the toxic activity as a secreted T6SS effector. These results indicate that a subset of MIX-effectors are found on mobile genetic elements and can be horizontally transferred between bacteria.

## Results

### *V*. *alginolyticus* 12G01 has two T6SSs

Upon analysis of its genomic sequences, the *V*. *alginolyticus* 12G01 strain was found to have two T6SSs that are similar to those previously reported for *V*. *alginolyticus* EPGS [25] and for *V*. *parahaemolyticus* RIMD 2210633 [20] (S1A Fig). VaT6SS1 contains two putative transcriptional regulators, Va01475 and Va01550, which are homologs of the *V*. *parahaemolyticus* T6SS1 positive regulators VP1407 and VP1391, respectively [20]. A homolog of the *V*. *parahaemolyticus* MIX-effector VP1388, Va01565, is found at the beginning of the VaT6SS1 gene cluster (S1A Fig) [16]. The VaT6SS2 gene cluster does not appear to encode any effectors or transcriptional regulators (S1A Fig).

As a preliminary step to characterize the T6SSs of *V*. *alginolyticus*, we first generated *V*. *alginolyticus* 12G01 derivative strains in which the T6SSs were inactivated by deletions in the genes encoding the necessary inner tube component Hcp of VaT6SS1 (Δ*hcp1*), VaT6SS2 (Δ*hcp2*), or both systems (Δ*hcp1*/Δ*hcp2*). Next, we tested whether inactivation of the VaT6SSs affected growth. To this end, we monitored the growth of the wild-type and Δ*hcp* strains in MLB media at 30°C by measuring the OD_600_ of the cultures over time. No difference in growth was detected (S1B Fig), indicating that the T6SSs do not affect *V*. *alginolyticus* growth.

### Both *V*. *alginolyticus* T6SSs have antibacterial activities and are differentially regulated by salinity and temperature

**Fig 1:**
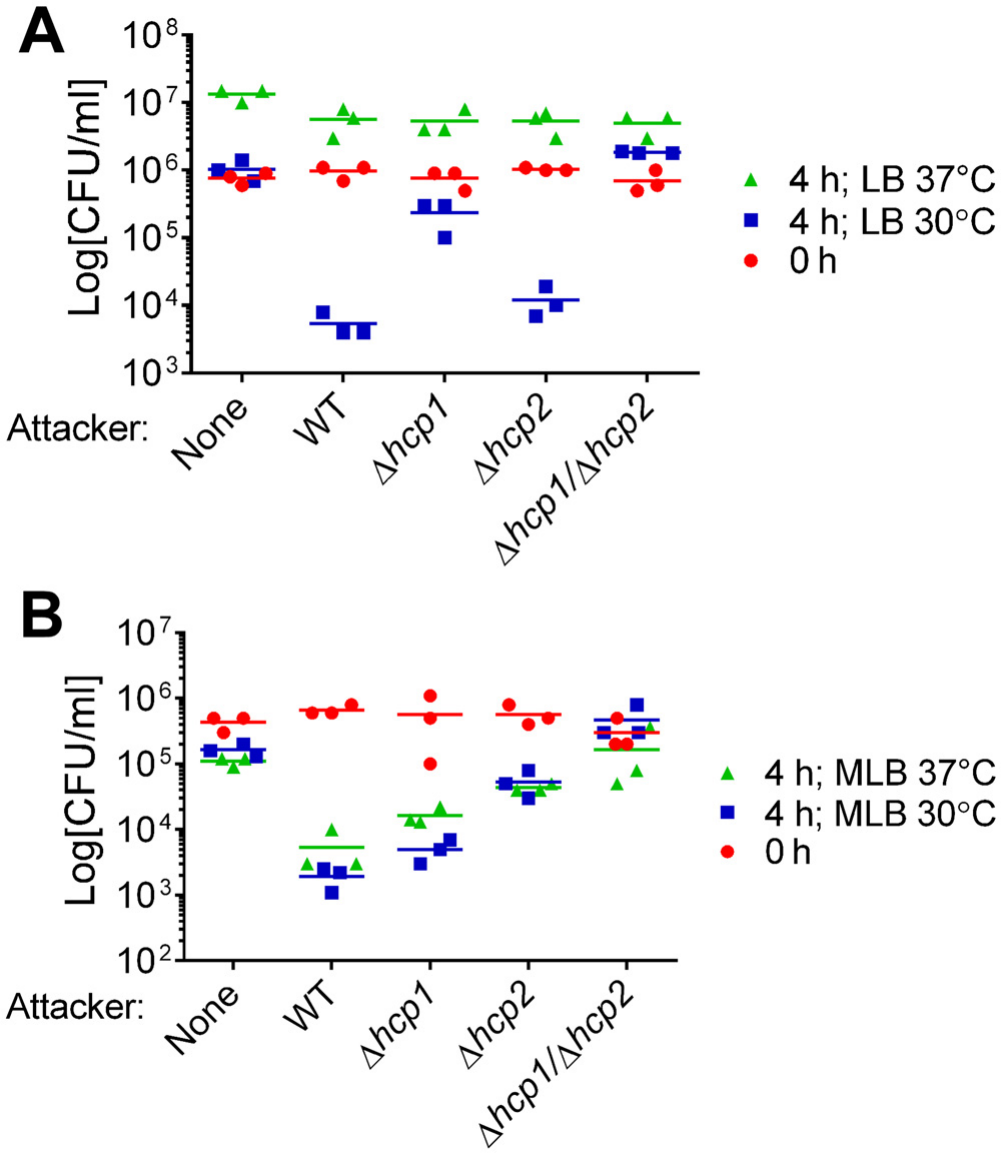
Antibacterial activities of *V*. *alginolyticus* T6SSs are differentially regulated by salinity and temperature. Viability of *E*. *coli* prey before (0h) and after (4h) co-culture with indicated *V*. *alginolyticus* attacker strains or alone. Co-cultures were incubated for 4 hours on (A) LB or (B) MLB agar plates at 30°C or 37°C. None = medium only. WT = wild-type.

We previously reported that VaT6SS1 mediates bacterial killing on LB agar plates at 30°C [16]. As *V*. *alginolyticus* is a marine bacterium that thrives during warm months under various conditions in the environment and the host [22,28], we set out to determine how environmental conditions such as salinity and temperature affect the activity of VaT6SS1. To this end, we monitored the viability of *E*. *coli* before and after co-culture with wild-type *V*. *alginolyticus*, a Δ*hcp1* derivative in which VaT6SS1 is inactive, or alone on LB or MLB plates (containing 1% and 3% NaCl, respectively), at 30°C or 37°C. When co-cultured at 37°C, *V*. *alginolyticus* was unable to kill *E*. *coli* on LB plates (Fig 1A), but it was able to kill *E*. *coli* on MLB plates (Fig 1B). Surprisingly, whereas deletion of *hcp1* largely abrogated the antibacterial toxicity of *V*. *alginolyticus* on LB at 30°C, it had only a marginal effect when co-cultures were grown on MLB plates at 30°C (Fig 1). This result suggested that there is another antibacterial determinant other than VaT6SS1 that can mediate interbacterial competition under high salt conditions.

We hypothesized that VaT6SS2 can also mediate antibacterial toxicity. To test our hypothesis, we repeated the *E*. *coli* competition assays with a Δ*hcp2* derivative in which VaT6SS2 is inactive and with a Δ*hcp1*/Δ*hcp2* derivative in which both VaT6SS1 and VaT6SS2 are inactive. Whereas deletion of *hcp2* had only a marginal effect on the antibacterial toxicity of *V*. *alginolyticus* on LB at 30°C, it had a considerable effect on MLB at 30°C (Fig 1). Consistent with these observations, the Δ*hcp1*/Δ*hcp2* derivative had no antibacterial toxicity under the tested conditions (Fig 1). These results indicated that both *V*. *alginolyticus* T6SSs possess antibacterial activities, yet they are active under different salinity and temperature conditions. VaT6SS1 is more active under low salt conditions (on LB plates), whereas VaT6SS2 is more active under high salt conditions (on MLB plates). Both T6SSs are active at 30°C, but only VaT6SS2 is also active at 37°C (Fig 1).

### Identification of *V*. *alginolyticus* T6SS2 effectors

After uncovering a role for VaT6SS2 in interbacterial competition, we next sought to identify the secreted effectors that mediate this antibacterial activity. To this end, we used comparative proteomics to find proteins that are secreted in a VaT6SS2-dependent manner. We used mass spectrometry (MS) to analyze the secretomes of *V*. *alginolyticus* Δ*hcp1* (with an active VaT6SS2) and Δ*hcp1*/Δ*hcp2* (with an inactive VaT6SS2) strains grown under VaT6SS2-inducing conditions (*i*.*e*. high salt media at 30°C) (see S1 Dataset). The strains used were deleted for *hcp1* to detect only proteins secreted by VaT6SS2. We identified 7 proteins that were differentially found in the supernatant of the Δ*hcp1* strain in which VaT6SS2 was active (Table 1). Va07588 is the VaT6SS2 tail tube secreted component VgrG2 and served to validate our VaT6SS2 secretome analysis (Table 1).

**Table 1:** *V*. *alginolyticus* VaT6SS2-dependent secreted proteins.

Role	Secreted Protein	Predicted domains/activity	Immunity	Homolog in *V*. *parahaemolyticus* ^a^	Sequence Coverage	Mean Spectral Count T6SS2- / T6SS2+	plgem p-value
**T6SS component**	Va07588	VgrG2		VPA1026	50.9%	0 / 77.7	2.69×10^−3^
**Effector**	Va16922	Nuclease	Va16927	NF ^b^	45.6%	0.3 / 24.3	4.13×10^−3^
	Va18287	RhsA, Nuclease	Va18282	VP1517	12.5%	0.3 / 40.7	1.18×10^−2^
	Va03175	LysM, Pore-forming	Va03170, Va03180	NF	18.8%	0 / 10.3	1.85×10^−2^
**Unknown**	Va03300	Lysozyme-like		NF	39.8%	0.3 / 10	2.24×10^−2^
	Va04801	Lipase_III		VP0626	45.3%	0.7 / 56.3	2.58×10^−3^
	Va13639	Unknown		NF	35.2%	0.7 / 14.7	5.73×10^−2^

^a^ *V*. *parahaemolyticus* RIMD 2210633

^b^ NF, not found.

**Fig 2:**
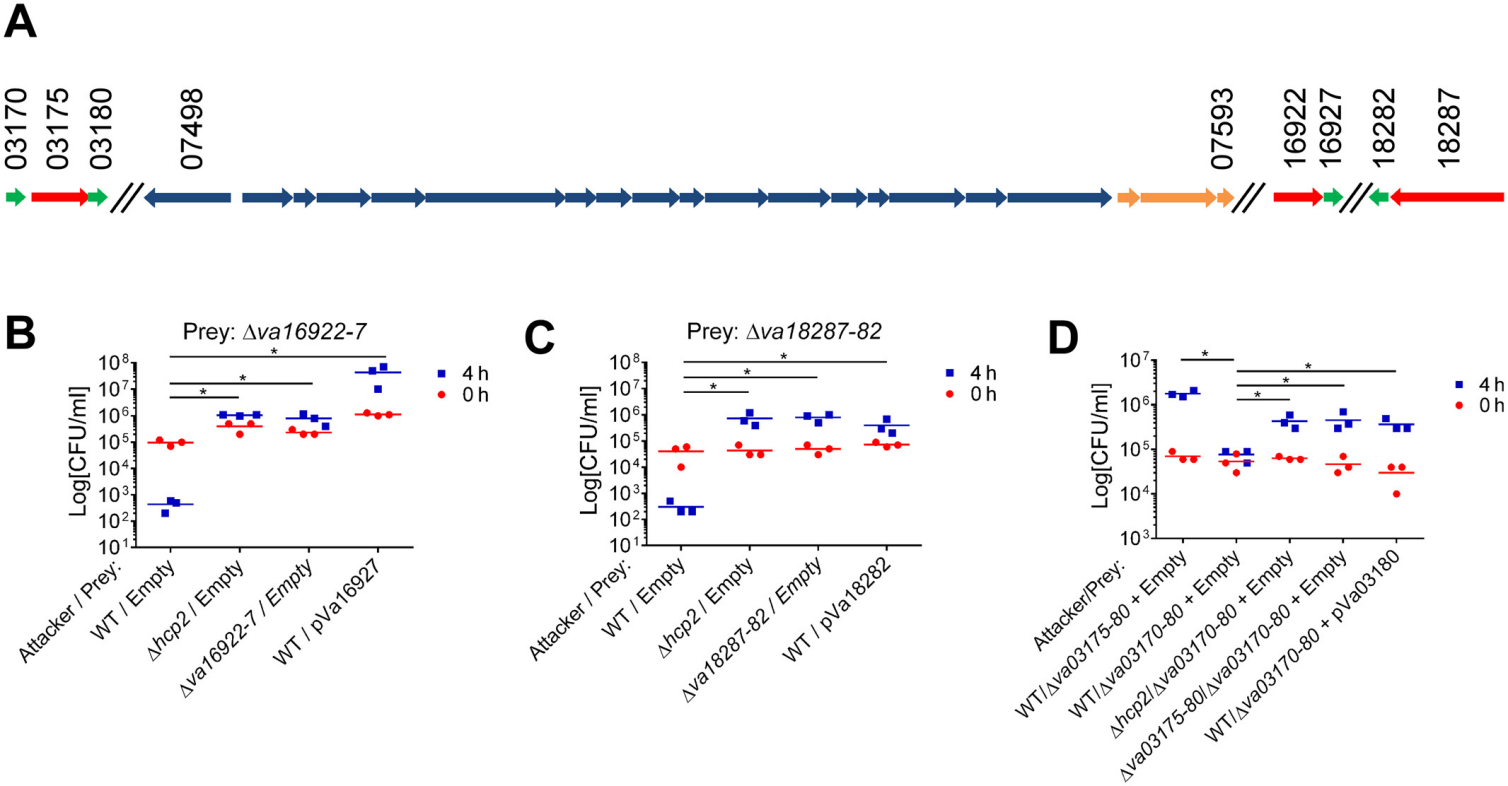
Va16922/Va16927, Va18287/Va18282, and Va03175/Va03170/Va03180 are VaT6SS2 effector/immunity pairs. (A) Schematic representation of the VaT6SS2 gene cluster and effector/immunity pairs. V12G01 locus numbers listed above. Effectors in red, Immunity in green, and tail tube components in orange. (B-D) Viability counts of prey strains containing an empty plasmid or a plasmid for the arabinose-inducible expression of the immunity protein before (0h) and after (4h) co-culture with the indicated attacker strains. Effector/immunity pairs tested were: (B) Va16922/Va16927, (C) Va18287/Va18282, and (D) Va03175/Va03170/Va03180. Asterisks mark statistical significance between sample groups at t = 4h by an unpaired, two tailed student’s t-test (p<0.05).

We predicted that three of the VaT6SS2 secreted proteins: Va16922, Va18287, and Va03175, were antibacterial effectors as they possess predicted nuclease (Va16922 and Va18287) or pore-forming colicin-like (Va03175) domains (according to HHPred analysis [29]) that can mediate antibacterial toxicity. Moreover, the genes encoding for these three proteins were immediately upstream of small open reading frames (ORFs) that could encode for their cognate immunity proteins (Va16927, Va18282, and Va03180, respectively). These three putative effector/immunity pairs were encoded outside of the VaT6SS2 gene cluster (Fig 2A). To test whether Va16922/7, Va18287/2, and Va03175/80 are VaT6SS2 effector/immunity pairs, we monitored the ability of a *V*. *alginolyticus* wild-type strain, which is immune against self-intoxication, to kill strains with deletions in the putative effector/immunity gene pairs. Indeed, the wild-type strain was able to kill strains with deletions in *va16922-7* and *va18287-2* when co-cultured under VaT6SS2 inducing conditions (Fig 2B and 2C), indicating that deletion of these bicistronic units resulted in loss of immunity against self-intoxication. However, a strain deleted for *va03175-80* was still immune against self-intoxication (Fig 2D), suggesting that either Va03175/80 are not an effector/immunity pair, or that there is an additional immunity gene. Using Va03180 as template, we performed a BLAST search to look for possible redundant immunity proteins in *V*. *alginolyticus* 12G01. We identified Va03170, encoded by the gene immediately upstream of the putative effector Va03175, as a homolog of Va03180 (65% identity). Therefore, we generated a strain deleted for the putative effector and the two homologous putative immunity genes, Δ*va03170-80*. As predicted, the wild-type strain was able to kill the Δ*va03170-80* strain when co-cultured under VaT6SS2 inducing conditions (Fig 2D), indicating that *va03170* and *va03180* encode for redundant immunity proteins against Va03175-medited toxicity. In all cases, inactivation of VaT6SS2 by deletion of *hcp2*, or deletion of the effector/immunity pair in the attacking strain, resulted in loss of self-intoxication indicating that the toxic effectors were delivered by VaT6SS2 and encoded within these bicistronic units. Moreover, exogenous expression of the putative immunity proteins from a plasmid in the prey strains deleted for the effector/immunity pairs restored immunity against self-intoxication (Fig 2). Importantly, expression of either Va03170 or Va03180 from a plasmid restored immunity against self-intoxication in the Δ*va03170-80* strain indicating they are indeed redundant immunity proteins against Va03175-mediated toxicity (Fig 2D and S2 Fig). Taken together, these results demonstrate that Va16922/7, Va18287/2, and Va03175/80/70, are effector/immunity pairs of VaT6SS2.

Three additional proteins, Va03300, Va04801, and Va13639, were secreted in a VaT6SS2-dependent manner (Table 1). However, we could not confidently determine whether they are VaT6SS2 antibacterial effectors or not, as either we did not identify an adjacent putative immunity gene (for Va13639), the short adjacent ORF was not associated with the gene encoding the secreted protein in other bacterial genomes and is thus not predicted to encode for its cognate immunity protein (for Va03300), or deletion of the adjacent ORF did not result in loss of immunity against self-intoxication and we did not find additional homologs of the putative immunity proteins encoded by *V*. *alginolyticus* 12G01 that could provide redundant immunity (for Va03300 and Va04801). Taken together, our results indicate that VaT6SS2 delivers at least three effectors into recipient cells to mediate antibacterial toxicity.

### Identification of *V*. *alginolyticus* T6SS1 effectors

To gain a more comprehensive understanding of the *V*. *alginolyticus* T6SS effector repertoires, we next set out to identify the effectors of VaT6SS1 using comparative proteomics. We were unable to detect VaT6SS1 activity under liquid growth conditions similar to those in which we saw VaT6SS1 antibacterial activity during competition experiments on agar plates (*i*.*e*. LB medium at 30°C) (S3 Fig). Nevertheless, we recently reported that H-NS, a bacterial histone-like nucleoid structuring protein, serves as a repressor of the *V*. *parahaemolyticus* VpT6SS1 and that its deletion results in activation of VpT6SS1 even under non-inducing conditions [27]. We hypothesized that H-NS can also act as a repressor of VaT6SS1 and that its deletion may lead to activation of the system. Indeed, when we used a *V*. *alginolyticus* strain deleted for *hns*, expression and secretion of Hcp1 that was endogenously tagged at the C-terminus with a FLAG tag (Hcp1-FLAG) were readily detected (S3 Fig), indicating that H-NS is a repressor of VaT6SS1 activity. This finding allowed us to analyze the VaT6SS1 secretome even when *V*. *alginolyticus* were grown in liquid.

With Δ*hns* strains, we used comparative proteomics to find proteins that are secreted only when VaT6SS1 is active. Again, we used MS to analyze the secretomes of *V*. *alginolyticus* Δ*hcp2/*Δ*hns* (with an active VaT6SS1) and Δ*hcp1*/Δ*hcp2/*Δ*hns* (with an inactive VaT6SS1) strains (see S2 Dataset). The strains used were also deleted for *hcp2* to detect only proteins secreted by VaT6SS1. We identified 6 proteins that were differentially found in the supernatant of the strain in which VaT6SS1 was active (Table 2). One of these proteins, Va01440, is the VaT6SS1 tail tube secreted component PAAR1 and served to validate our VaT6SS1 secretome analysis.

**Table 2:** *V*. *alginolyticus* VaT6SS1-dependent secreted proteins.

Role	Secreted Protein	Predicted domains/activity	Immunity	Homolog in *V*. *parahaemolyticus* ^a^	Sequence Coverage	Mean Spectral CountT6SS1- / T6SS1+	Plgemp-value
**T6SS component**	Va01440	PAAR1		VP1415	37.9%	0.3 / 14	3.00×10^−3^
**Effector**	Va16152	MIX IV, Pore-forming	Va16147	NF ^b^	26.4%	0.7 / 17	9.00×10^−3^
	Va01565	MIX I	Va01560	VP1388	28.1%	0 / 7	1.36×10^−2^
	Va01435	LytM, PG-hydrolase, Lysozyme-like	Va01430	NF	37.6%	0 / 15	3.69×10^−3^
**Unknown**	Va01555	OmpA_C		VP1390	55.3%	0.3 / 71	1.74×10^−3^
	Va17542	VopQ		VP1680	36.8%	1.7 / 57.7	3.57×10^−4^

^a^ *V*. *parahaemolyticus* RIMD 2210633

^b^ NF, not found.

**Fig 3:**
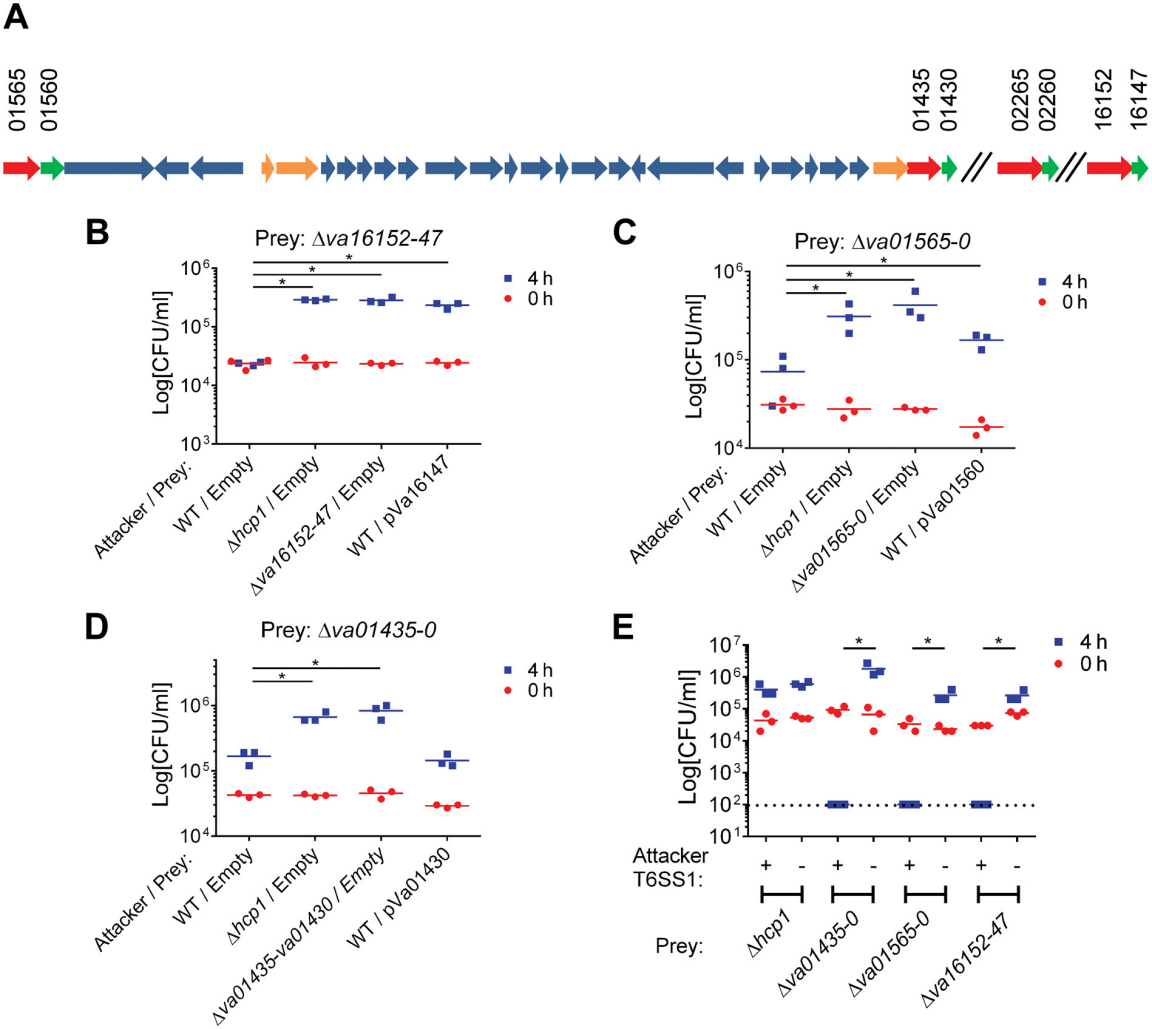
Va16152/Va16147, Va01565/Va01560, and Va01435/Va01430 are VaT6SS1 effector/immunity pairs. (A) Schematic representation of the VaT6SS1 gene cluster and effector/immunity pairs. V12G01 locus numbers listed above. Effectors in red, Immunity in green, and tail tube components in orange. (B-D) Viability counts of prey strains containing an empty plasmid or a plasmid for the arabinose-inducible expression of the immunity protein before (0h) and after (4h) co-culture with the indicated attacker strains. Effector/immunity pairs tested were: (B) Va16152/Va16147, (C) Va01565/Va01560, and (D) Va01435/Va01430. (E) Viability counts of prey strains before (0h) and after (4h) co-culture with *V*. *alginolyticus* 12G01 Δ*hcp2*/Δ*hns* (T6SS1+) or Δ*hcp1/*Δ*hcp2*/Δ*hns* (T6SS1-) strains. Dashed line marks the assay detection limit. Asterisks mark statistical significance between sample groups at t = 4h by an unpaired, two tailed student’s t-test (p<0.05).

As predicted by the presence of the MIX motif, two of the identified secreted proteins, Va16152 and Va01565, were previously classified by us as putative T6SS MIX-effectors [16]. Va16152 contains an N-terminal MIX motif belonging to the MIX IV clan and a C-terminal pore-forming colicin-like domain (according to HHPred analysis [29]), and is encoded outside of the VaT6SS1 gene cluster. Va01565, which is encoded at the beginning of the VaT6SS1 gene cluster, contains a MIX motif belonging to the MIX I clan and is a homolog of the *V*. *parahaemolyticus* MIX-effector VP1388 [16]. Another VaT6SS1 secreted protein, Va01435, is encoded at the end of the VaT6SS1 gene cluster and is predicted to contain an N-terminal LysM peptidoglycan-binding domain followed by a peptidoglycan (PG) hydrolase domain and a lysozyme-like domain (according to HHPred analysis [29]). Moreover, the genes encoding for these three proteins were immediately upstream of small ORFs that could encode for their cognate immunity proteins (Va16147, Va01560, and Va01430, respectively) (Fig 3A). To test whether Va16152/47, Va01565/0, and Va01435/0 are VaT6SS1 effector/immunity pairs, we monitored the ability of a *V*. *alginolyticus* wild-type strain to kill strains with deletions in the putative effector/immunity gene pairs. As shown in Fig 3B–3D, the wild-type strain was able to inhibit the growth of strains with deletions in *va16152-47*, *va01565-0* and *va01435-0* when co-cultured under VaT6SS1 inducing conditions, and inactivation of VaT6SS1 by deletion of *hcp1* or deletion of the effector/immunity pairs in the attacking strains resulted in increased growth of the prey strains. Moreover, exogenous expression of the putative immunity proteins Va16147 and Va01560 from a plasmid, but not of Va01430, also resulted in increased growth of the prey strains deleted for the cognate effector/immunity pairs. It is possible that the inability of the plasmid encoding for Va01430 to complement the deletion resulted from poor expression of Va01430 under the tested conditions.

We were surprised by the minor deleterious effect of the wild-type attacker strains on the effector/immunity deletion strains, as we expected that the VaT6SS1 would mediate bacterial killing based on the toxic effects observed for VaT6SS1 when *E*. *coli* was used as prey (Fig 1). We reasoned that perhaps the toxic effects were minor because we failed to properly activate the VaT6SS1 under the tested conditions and thus were not observing the full toxic effect of the individual effectors. To test whether this is the case, we used the Δ*hcp2/*Δ*hns* strain (with a constitutively active VaT6SS1) as an attacker strain in competition assays with the effector/immunity deletion strains. As predicted, upon de-repression of VaT6SS1 by deleting H-NS the attacking strain was able to kill all tested effector/immunity deletion strains (Fig 3E). This killing was VaT6SS1-mediated as a Δ*hcp1*/Δ*hcp2/*Δ*hns* attacking strain (with an inactive VaT6SS1) was not toxic to the prey strains. Notably, the constitutive activation of VaT6SS1 in the attacking strain did not result in bacterial toxicity simply because of over-expression or delivery of effectors, as it was not toxic to a Δ*hcp1* prey strain that did not have an active VaT6SS1 but still had all of the genes encoding for the putative immunity proteins (Fig 3E).

Two additional proteins, Va01555 and Va17542, were secreted in a VaT6SS1-dependent manner in our comparative proteomics analysis (Table 2). However, we concluded that they were most likely not *bona fide* antibacterial VaT6SS1 effectors. Va01555 is a homolog of the *V*. *parahaemolyticus* VP1390 which we previously identified as secreted by VpT6SS1 but ruled out as an antibacterial effector because it had no detectable immunity protein [16]. Va17542 is a homolog of the *V*. *parahaemolyticus* VopQ, a virulence effector protein of the Type III Secretion System 1 (T3SS1) [30–32]. It is possible that the detection of Va17542 in our secretome was an artifact resulting from hyper-activation of T3SS1 by deletion of *hns* [33]. Taken together with our previous identification of the VaT6SS1 MIX-effector Va02265 [16], our results indicate that VaT6SS1 delivers at least four effectors, three of which are MIX-effectors, into recipient cells to mediate antibacterial toxicity.

### MIX-effectors transmitted horizontally between *Vibrios*

**Fig 4:**
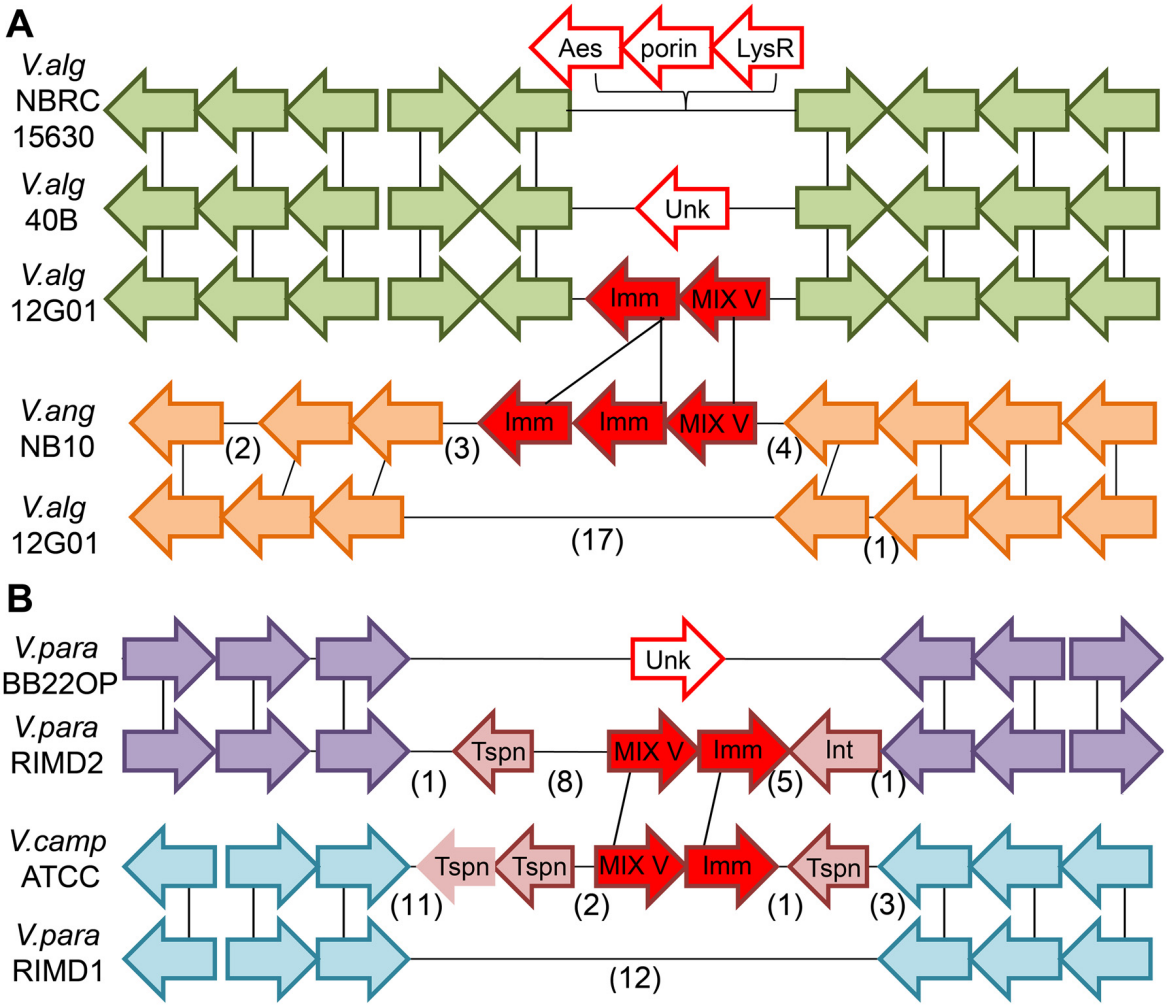
Mobility of MIX V-effector/immunity cassettes. Genome neighborhoods are illustrated using arrows to indicate gene orientation, with gene correspondence between species indicated by black vertical lines and omitted genes indicated in parentheses. (A) The “orphan” Duf2235-containing MIX V-effector/immunity pair (*va02265*/*0*, filled red arrows) encoded by the *V*. *alginolyticus* 12G01 scaffold (*V*.*alg*12G01) falls within a conserved gene neighborhood present in other *V*. *alginolyticus* strains (green arrows). The orphan pair is absent from the alternate *V*. *alginolyticus* strain genomes and is replaced by an alternate cassette of 3 different genes (open red arrows) in *V*. *alginolyticus* NBRC 15630 = ATCC 17749 chromosome 2 (*V*.*alg*NBRC15630) and an unknown gene in *V*. *alginolyticus* 40B scaffold (*V*.*alg*40B). A homologous Duf2235-containing MIX V-effector with a duplicated immunity gene (filled red arrows) can be found in a more distant *Vibrio* strain: *V*. *anguillarum* NB10 chromosome 2 (*V*.*ang*NB10) in an alternate gene neighborhood (orange arrows). (B) The Colicin DNase-containing MIX V-effector/immunity pair (*vpa1263*/*vti2*, filled red arrows) encoded by *V*. *parahaemolyticus* RIMD 2210633 chromosome 2 (*V*.*para*.RIMD2) belongs to a genetic island that includes a transposon and phage integrase (pink arrows). The island is not present in similar strains such as *V*. *parahaemolyticus* BB22OP chromosome 2 (*V*.*para*.BB22OP) that retains the surrounding conserved gene neighborhood (purple arrows). A homologous Colicin DNase-containing MIX V-effector/immunity cassette (filled red arrows) is present in a more distant *Vibrio* strain, *Vibrio campbellii* ATCC BAA-1116 (*V*.*camp*ATCC), in an alternate gene neighborhood (cyan arrows). Neighborhoods include genes (from left to right): *N646_ 3835*—*N646_3824* from *V*.*alg*.NBRC15630, *V12G01_02235*—*V12G01_02286* and alternate genes *V12G01_08143—V12G01_08023* from *V*.*alg*.12G01, *VMC_26590*—*VMC_26680* for *V*.*alg*40B, *VANGNB10_cII03835—VANGNB10_cII03824* for *V*.*ang*NB10, *VPBB_A1148—VPBB_A1154* for *V*.*para*.BB22OP, *VPA1250—VPA1273* and alternate genes *VP0094—VP0077* from *V*.*para*.RIMD, and *VIBHAR_00561—VIBHAR_00534* from *V*.*camp*ATCC. Tspn = transposase, Int = integrase, Imm = immunity, Unk = unknown.

We next examined whether the VaT6SS1 effector/immunity pairs that we identified in the 12G01 strain were also found in other *V*. *alginolyticus* strains. Homologs of the effector Va01435, as well as of the two MIX-effectors Va01565 and Va16152, and their cognate immunity proteins were encoded in the genomes of other sequenced *V*. *alginolyticus* strains (*i*.*e*. NBRC 15630, E0666, and 40B) in the same synteny as in strain 12G01. However, the bicistronic unit encoding the MIX-effector/immunity pair Va02265/0 was not found in the genomes of other sequenced *V*. *alginolyticus* strains (Fig 4A), suggesting that it was recently acquired by the 12G01 strain. Interestingly, we found bicistronic units encoding homologs of the Va02265/0 MIX-effector/immunity cassette in genomes of other *Vibrio* species (*e*.*g*. *V*. *anguillarum* NB10), albeit at a synteny distinct from the one it had in *V*. *alginolyticus* 12G01 (Fig 4A). These results suggested that this MIX-effector/immunity cassette might be transmitted horizontally between *Vibrio* species.

In our previous work, we reported that MIX-effectors group into five distinct clans named MIX I-V based on the sequences of their MIX-containing regions [16]. The *V*. *alginolyticus* MIX-effector Va02265 belongs to the MIX V clan and does not neighbor other T6SS components on the genome [16]. Therefore, we classified it as an "orphan" MIX-effector. To test whether other MIX V-effectors are "orphan", we next examined the genome neighborhoods of genes encoding other MIX V-effectors. Remarkably, we found that most members of the MIX V clan are “orphan” MIX-effectors that do not neighbor any other T6SS component (Only 1 out of 124 identified MIX V-effectors was found to neighbor T6SS components; 35 out of the 124 remain uncertain as not all neighboring genes were identified; See S3 Dataset and S1 File). Furthermore, MIX V clan members were only found in marine γ-proteobacteria, with the vast majority distributed among *Vibrionales* (117), and a few found in *Alteromonadales* (4), *Aeromonadales* (2), and *Oceanospirillaales* (1) (S4 Fig). We also noticed that some MIX V members are encoded adjacent to transposable elements such as transposases and integrases. Thus, we hypothesized that MIX-effector that belong to the MIX V clan are mobile and can be shared between marine bacteria. In support of this notion, the “orphan” MIX V-effector that we previously identified in *V*. *parahaemolyticus* RIMD 2210633, VPA1263 [16], is encoded within the *V*. *parahaemolyticus* island-6 (VPaI-6; *vpa1254-vpa1270*) that contains a transposase and an integrase. This VPaI-6 was suggested to be a mobile element acquired by pandemic strains [34]. The bicistronic unit encoding VPA1263 and its cognate immunity Vti2 [16] can be found in distinct locations on the genomes of other *Vibrios* (*e*.*g*. *Vibrio campbelli* ATCC BAA-1116) flanked by transposase genes (Fig 4B).

Furthermore, MIX V members can be located on plasmids. In *Aliivibrio salmonicida*, one MIX V member (VSAL_p840_46) is encoded adjacent to an IS insertion element (*VSAL_p840_45*) on one of four plasmids that also includes T6SS components (*i*.*e*. VgrG: *VSAL_p840_36* and Hcp: *VSAL_p840_35*) and viral conjugative transfer genes (*VSAL_p840_1* –*VSAL_p840_21*). A second MIX V-effector that is more closely related to the plasmid copy than to other MIX V members resides in the chromosome (*VSAL_I0031*) near a noted transposase (*VSAL_I0029*). These findings further support our hypothesis that “orphan” MIX-effectors belonging to the MIX V clan are mobile T6SS effectors that can move between Gram-negative marine bacteria via horizontal gene transfer and be used as effectors by MIX-secreting T6SSs.

**Fig 5:**
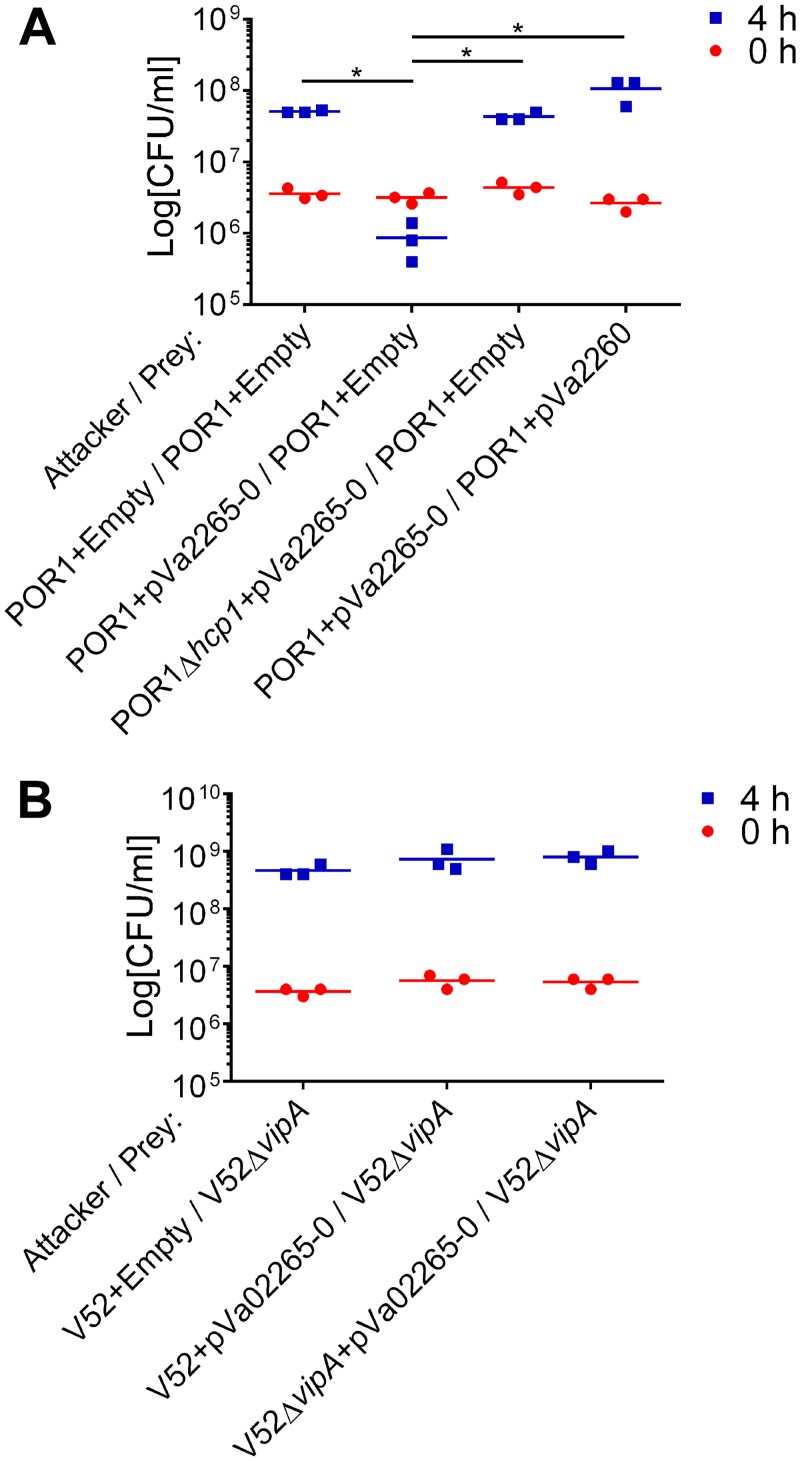
*V*. *parahaemolyticus* T6SS1 can deliver an “orphan” *V*. *alginolyticus* MIX V-effector. Viability counts of prey strains before (0h) and after (4h) co-culture with indicated attacker strains. (A) *V*. *parahaemolyticus* POR1 prey strains containing an empty plasmid or a plasmid for the arabinose-inducible expression of the immunity protein Va02260 (pVa02260) were co-cultured with POR1 or POR1Δ*hcp1* attacker strains containing an empty plasmid or a plasmid for the arabinose-inducible expression of the Va02265/Va02260 effector/immunity pair (pVa02265-0). (B) *V*. *cholerae* V52Δ*vipA* prey strains were co-cultured with V52 or V52Δ*vipA* attacker strains containing an empty plasmid or a plasmid for the arabinose-inducible expression of the Va02265/Va02260 effector/immunity pair. Asterisks mark statistical significance between sample groups at t = 4h by an unpaired, two tailed student’s t-test (p<0.05).

Whereas previous reports demonstrated that homologous effectors from different species can be secreted by T6SSs [35], the possibility that bacteria can use their T6SSs to secrete newly acquired effectors which are not naturally encoded in their genome has not been addressed. To directly test whether bacteria can use a newly acquired mobile MIX V-effector to increase their competitive fitness, we used the *V*. *alginolyticus* MIX V-effector/immunity pair Va02265/0 and asked whether a *V*. *parahaemolyticus* that contains a MIX-secreting T6SS (VpT6SS1) but no homologs of Va02265 can use Va02265 as a T6SS effector to gain competitive advantage over its parental kin (which are otherwise immune against self-intoxication). Indeed, the *V*. *parahaemolyticus* POR1 strain was able to kill a POR1 parental prey when the Va02265/0 effector/immunity cassette was introduced and expressed from a plasmid in the attacking strain (Fig 5A). This Va2265-mediated self-intoxication was dependent on VpT6SS1 activity, as an attacker strain expressing the effector/immunity cassette that had an inactive VpT6SS1 (POR1Δ*hcp1*) was no longer able to kill the parental prey. Moreover, expression of the Va02260 immunity protein from a plasmid in the parental prey strain resulted in immunity against the Va02265-mediated intoxication. Thus, these results demonstrate that an “orphan” MIX-effector belonging to the MIX V clan can be used by another *Vibrio* strain as a T6SS effector and provide competitive advantage. However, it appears that there is some degree of specificity towards T6SSs, as the *V*. *cholerae* V52 strain was unable to use Va02265 as an antibacterial effector in a self-intoxication assay (Fig 5B) under conditions in which this strain mediated T6SS-dependent killing of *E*. *coli* [36].

## Discussion

In this work, we used genetic and proteomic analyses to characterize the environmental conditions that activate the two T6SSs found in the marine pathogen *V*. *alginolyticus*, identify their functions, and determine their effector repertoires. We found that both T6SSs mediate interbacterial competition although they are active under different salinity and temperature conditions, suggesting they are utilized by this bacterium in different environments.

Surprisingly, even though the *V*. *alginolyticus* T6SS gene clusters are similar to those of *V*. *parahaemolyticus* in terms of gene content and organization, and both bacteria reside in similar habitats, the regulation of their T6SSs differs. For example, whereas the *V*. *parahaemolyticus* VpT6SS1 is active under high salt conditions, it appears that its *V*. *alginolyticus* counterpart, VaT6SS1, is active under low salt conditions. Moreover, in our previous studies we were unable to detect antibacterial activity for VpT6SS2 due to repression of the system by surface-sensing activation under our bacterial competition assay conditions [20]. However, the results shown here indicate that VaT6SS2 is not inhibited by surface-sensing as antibacterial activity was readily detectable in competition assays performed on agar plates. This differential regulation of the *V*. *parahaemolyticus* and *V*. *alginolyticus* T6SSs allowed us to identify the antibacterial activity of VaT6SS2 and its effector repertoire.

In this work we identified six new T6SS effectors in *V*. *algnolyticus* 12G01. While we are currently investigating their biochemical activities, our results demonstrate that all six effectors mediate antibacterial toxicities. The presence of antibacterial T6SS effectors in bicistronic units together with genes that encode for their cognate immunity proteins is well documented [7,11,16,37]. Indeed, we showed that the proteins encoded downstream of the six effectors that were secreted in a T6SS-dependent manner in our comparative proteomics analyses do provide immunity against T6SS-mediated intoxication. Taken together with the various putative toxin domains found in the six T6SS-secretd proteins, we conclude that they are antibacterial T6SS effectors.

Based on the three VaT6SS2 effectors that we identified in this work, we hypothesize that the *V*. *parahaemolyticus* VpT6SS2 also mediates antibacterial activity under conditions we have yet to uncover. This hypothesis is supported by the presence of a close homolog to the VaT6SS2 effector Va18287, a member of the Rhs class of T6SS effectors, in the *V*. *parahaemolyticus* genome (Table 1) [12]. The *V*. *parahaemolyticus* homolog, VP1517, contains an RhsA domain and a predicted C-terminal nuclease domain of the HNH/ENDO VII superfamily which is often found in bacterial toxins and could thus serve as an antibacterial T6SS effector [15].

Notably, the impact of our previous discovery of the MIX motif, which enabled us to identify hundreds of effectors belonging to the MIX-effector class in various bacterial species [16], is further underscored in this work. While we predicted the presence of three MIX-effectors in *V*. *alginolyticus* 12G01, we only found two secreted MIX-effectors in our comparative proteomics analysis (Va01565 and Va16152). Thus, the third MIX-effector, Va02265, would not have been identified if not for the presence of the MIX motif in its sequence, as it was not encoded close to other T6SS components on the genome (and therefore labeled as an "orphan" MIX-effector). Furthermore, our finding that the *V*. *alginolyticus* VaT6SS1 can secrete three MIX-effectors that belong to different MIX clans (Va01565 to MIX I, Va16152 to MIX IV, and Va02265 to MIX V) implies that T6SSs have a certain degree of freedom in the MIX-effectors they can secrete.

Our observation that most members of the MIX V clan are "orphan" MIX-effectors that often neighbor transposable elements led us to hypothesize that they are mobile and shared between marine bacteria. Remarkably, Borgeaud *et*. *al*. recently reported that the T6SS is part of the competence regulon in *V*. *cholerae* [38]. They showed that T6SS-mediated killing allows *V*. *cholerae* to uptake the released DNA of the lysed bacterial competitor and incorporate it into its own DNA, thus fostering horizontal gene transfer and driving evolution [38]. It is therefore compelling to speculate that similar mechanisms are found in other marine bacteria, and that bacteria can use their T6SSs to prey on DNA from their competitors and acquire new mobile MIX V-effector/immunity cassettes that will provide increased fitness in future competitions as they diversify their T6SS effector repertoires.

Another mechanism that drives evolution of virulence factors is the presence of non-integrated conjugative plasmids like in *Aliivibrio salmonicida* LFI1238. Codon usage analysis showed that the genomic chromosomal copy of the MIX V-effector in *Aliivibrio* is more closely related to the plasmid copy than to the genome background [39], suggesting the chromosomal gene that neighbors transposases originated from the plasmid. The observed high occurrence of transposable elements in the *Aliivibrio salmonicida* LFI1238 genome is thought to mediate this gene transfer and represent a mechanism for driving diversity in the chromosome. It is therefore possible that similar mechanisms are found in other *Vibrios* to enable horizontal gene transfer of mobile MIX V-effectors.

We found that MIX V-effectors are only present in marine bacteria, mostly in members of the *Vibrionales* family. These bacteria can interact with each other in the same aquatic habitats, thus providing access to various MIX V-effectors from competing species. A similar phenomenon was recently reported in *Xanthomonads*, where Tn3-like transposons play a role in spreading virulence effectors of the T3SS via horizontal gene transfer [40]. In conclusion, we propose that mobile MIX V-effectors serve as an environmental reservoir of polymorphic antibacterial toxins that can be shared between marine bacteria via horizontal gene transfer and used to enrich the versatility of T6SS effector repertoires, thus increasing competitive fitness.

## Methods

### Strains and media

The *Vibrio alginolyticus* 12G01 strain and the *Vibrio parahaemolyticus* RIMD 2210633 derivative strain POR1 (RIMD 2210633 Δ*tdhAS*) [41] and their derivatives were routinely cultured in Marine Luria-Bertani (MLB) broth (Luria-Bertani broth containing 3% sodium chloride) or on Marine minimal media (MMM) agar [42] at 30°C. The *Vibrio cholerae* V52 strain and its derivative V52Δ*vipA* (a gift from Dr. J. Mekalanos, Harvard Medical School) [4,43] were routinely cultured in LB broth at 37°C. *E*. *coli* DH5α and S17-1(λ pir) were routinely cultured in 2×YT broth at 37°C. The medium was supplemented with kanamycin or chloramphenicol where necessary. Arabinose was added to solid or liquid media at a final concentration of 0.1% (w/v) when induction of expression from a plasmid was required.

### Plasmids and construction of deletion and knock-in strains

For ectopic expression of *va01560*, *va01430*, *va16147*, *va03180*, *va03170*, *va18282*, and *va16927*, the genes' coding sequences were amplified and cloned into the MCS of the arabinose-inducible expression vector pBAD33 (Invitrogen) containing chloramphenicol resistance. For ectopic expression of the effector/immunity gene pair, the coding regions of effector and immunity genes *va02265-va02260* were amplified together, including the stop codons, and cloned into the MCS of the pBAD/*Myc*-His vector (Invitrogen) in which the antibiotic resistance was changed from ampicillin to kanamycin. The resulting plasmids were conjugated into *V*. *alginolyticus*, *V*. *cholerae*, or *V*. *parahaemolyticus* using tri-parental mating. For the generation of in-frame *Vibrio* deletion strains, the nucleotide sequences 1 kb upstream and 1 kb downstream of the effector/immunity pairs, the *hcp* genes (*va01540* and *va07583* are *hcp1* and *hcp2*, respectively) or *hns* (*va20201*) were amplified and cloned together into pDM4, a Cm^R^OriR6K suicide plasmid. For the generation of the C-terminal FLAG tagged Hcp1 strain, a C-terminal FLAG tagged version and the nucleotide sequences 1 kb downstream of the *V*. *alginolyticus hcp1* were amplified and cloned together into pDM4. The resulting pDM4 plasmid was conjugated into *V*. *alginolyticus* from *E*. *coli* S17-1(λ pir) and transconjugants were selected on media containing 25 μg/ml chloramphenicol. Bacteria were counter-selected by growing on media containing 15% sucrose. Deletions and insertions were confirmed by PCR.

### Bacterial growth assays

Assay was performed as previously described [20] and was repeated twice with similar results. Results of a representative experiment are shown.

### Hcp-FLAG expression and secretion

*Vibrio* strains were grown overnight in MLB. Cells were washed and re-suspended in 5 ml of LB media. Cultures were incubated with agitation for 5 hours at 30°C. Expression and secretion were determined by immunoblot as previously described [20] with anti-FLAG antibodies (Sigma Aldrich). Equal loading of total protein lysates was confirmed by analysis of representative bands using Ponceau S staining of the immunoblot membrane.

### Bacterial competition

Bacterial strains were grown over-night in MLB (*V*. *alginolyticus* and *V*. *parahaemolyticus*), LB (*V*. *cholerae*), or 2xYT (*E*. *coli*). Bacterial cultures were mixed and spotted on LB or MLB plates as previously described [20]. CFU of the prey spotted at t = 0h were determined by plating 10-fold serial dilutions on selective media plates. Bacterial spots were harvested from plates after 4 hours incubation and the CFU of the surviving prey cells were determined. Assays were repeated at least twice with similar results, and results of a representative experiment are shown.

### Mass-spectrometry analyses

For analysis of the VaT6SS2 secretome, *V*. *alginolyticus* cultures of Δ*hcp1* (with an active VaT6SS2; T6SS2+) and Δ*hcp1*/Δ*hcp2* (with an inactive VaT6SS2; T6SS2-) strains were grown in triplicate in 50 ml MLB media at an initial OD_600_ = 0.54 for 5 h at 30°C. For analysis of the VaT6SS1 secretome, *V*. *alginolyticus* cultures of Δ*hcp2/*Δ*hns* (with an active VaT6SS1; T6SS1+) and Δ*hcp1*/Δ*hcp2/*Δ*hns* (with an inactive VaT6SS1; T6SS1-) strains were grown in triplicate in 50 ml LB media at an initial OD_600_ = 0.18 for 5 h at 30°C. Media were collected and proteins precipitated as previously described [44]. Protein samples were run 10 mm into the top of an SDS-PAGE gel, stained with Coomassie Blue, and excised. Overnight digestion with trypsin (Promega) was performed after reduction and alkylation with DTT and iodoacetamide (Sigma—Aldrich). The resulting samples were analyzed by tandem MS using either a QExactive or Orbitrap Elite mass spectrometer (Thermo Electron) coupled to an Ultimate 3000 RSLC-Nano liquid chromatography system (Dionex). Peptides were loaded onto either a 180 μm i.d., 15-cm long, self-packed column containing 1.9 μm C18 resin (Dr. Maisch, Ammerbuch, Germany) or a 75 μm i.d., 50-cm long Easy Spray column (Thermo) and eluted with a gradient of either 0–28% buffer B for 40 min or 0–28% buffer B for 60 min. Buffer A consisted of 2% (v/v) acetonitrile (ACN) and 0.1% formic acid in water. Buffer B consisted of 80% (v/v) ACN, 10% (v/v) trifluoroethanol, and 0.08% formic acid in water. To ensure accurate Label-free quantification, control and experiment samples (*i*.*e*. T6SS1+/T6SS1- or T6SS2+/T6SS2-) were run on the same column with the same gradient using the same instrument. The mass spectrometer acquired up to 10 fragment spectra for each full spectrum acquired Raw MS data files were converted to peak list format using ProteoWizard msconvert (version 3.0.3535) [45]. The resulting files were analyzed using the central proteomics facilities pipeline (CPFP), version 2.1.0 [46,47]. Peptide identification was performed using the X!Tandem [48] and open MS search algorithm (OMSSA) [49] search engines against a database consisting of *V*. *alginolyticus* 12G01 sequences from UniProt KnowledgeBase, with common contaminants and reversed decoy sequences appended [50]. Fragment and precursor tolerances of 20 ppm and 0.1 Da were specified, and three missed cleavages were allowed. Carbamidomethylation of Cys was specified as a fixed modification, and oxidation of Met was specified as a variable modification. Label-free quantitation of proteins across samples was performed using SINQ normalized spectral index software [51]. To identify statistically significant differences in protein amount between T6SS1+/T6SS1- and T6SS2+/T6SS2− strains, SINQ quantitation results for three biological replicates per strain were processed using the power law global error model (PLGEM) package in R [52,53]. Protein identifications were filtered to an estimated 1% protein false discovery rate (FDR) using the concatenated target-decoy method [50]. An additional requirement of two unique peptide sequences per protein was imposed, resulting in a final protein FDR<1%. Spectral index quantitation was performed using peptide-to-spectrum matches (PSMs) with a q-value≤0.01, corresponding to a 1% FDR rate for PSMs. The datasets of tandem MS results were uploaded to the MassIVE repository (http://massive.ucsd.edu/ProteoSAFe/status.jsp?task=f4d7613b8ee2414985a8e0bbfcf905fe; MassIVE ID: MSV000078946).

### Identification of MIX V-effectors

To identify MIX V-effectors, we queried the nr database from NCBI (Feb 24, 2015) with the N-terminal sequence of VPA1263 (gi| 28901118, 1–320) that includes MIX V using PSI-BLAST [54] with default values (E-value cutoff 0.005, 5 iterations). We limited the resulting hits to include only refseq sequences from complete genomes, and clustered the bounded sequence hits using CLANS [55]. Sequences that cluster together with VPA1263 and cover the MIX V sequence motifs are represented in S3 Dataset. The Va02265 MIX V sequence was detected by PSI-BLAST, but is omitted from the resulting database, as it is not part of refseq. Taxonomic distributions of the corresponding sequences were generated with batch entrez on the NCBI website.

To locate the genome neighborhoods of MIX V effectors, we located their NCBI gene identifiers (gi) among nucleotide records of completely sequences genomes. We noted the gis corresponding to protein sequences located adjacent to MIX V (+/- 3 genes). The completely sequenced genomes represent various stages of assembly completeness linking shorter contigs into ordered genomes. For those MIX V that reside near the ends of shorter contigs, we designated any missing neighbors with an “x” (see S3 Dataset and S1 File for additional information). We collected all protein sequences corresponding to MIX V-effectors and their neighbors and defined the domain content from the COG database using batch CD-search [56] on the NCBI server (default cutoff E-value 0.01). To determine whether a MIX V gene neighbors T6SS components, we manually searched identified domains for those that correspond to the 13 core T6SS components, as previously identified by Boyer et al. [17].

### MIX V gene organization

Protein sequence databases were generated for each species using coding sequence collected from NCBI nucleotide records for *Vibrio parahaemolyticus* RIMD 2210633 chromosome 1 (NC_004603.1) and chromosome 2 (NC_004605.1), *Vibrio parahaemolyticus* BB22OP chromosome 1 (NC_019955.1) and chromosome 2 (NC_019971.1), *Vibrio alginolyticus* 12G01 scaffold (CH902589.1), *Vibrio alginolyticus* NBRC 15630 = ATCC 17749 chromosome 1 (NC_022349.1) and chromosome 2 (NC_022359.1), *Vibrio alginolyticus* 40B scaffold (ACZB01000054.1), *Vibrio anguillarum* NB10 chromosome 2 (LK021129), and *Vibrio campbellii* ATCC BAA-1116 chromosome 1 (NC_022269.1). To define gene correspondence between species, MIX V and surrounding genes from *Vibrio parahaemolyticus* RIMD 2210633 and *Vibrio alginolyticus* 12G01 were used as BLAST queries against each database, keeping only top hits (E-value cutoff 0.001). Conserved ordering of top BLAST hits in the chromosomes (of scaffold) was considered to define gene synteny between the various species. Domain annotations were defined according to NCBI conserved domain database (cdd).

## Supporting Information

S1 Fig T6SS deletions do not affect *V*. *alginolyticus* growth. (A) Organization of the *V*. *alginolyticus* 12G01 T6SS gene clusters. Direction of gene transcription is represented by arrows. V12G01 locus numbers listed above; T6SS component name according to accepted nomenclature shown below. (B) Growth of *V*. *alginolyticus* 12G01 and derivative strains in MLB at 30°C shown as OD_600_ measurements. Data are mean ± SD, n = 3. WT = wild-type.
(TIF)

S2 Fig Va03170 and Va03180 are redundant immunity proteins against Va03175 toxicity. Viability counts of Δ*va03170-80* prey strain containing an empty plasmid or a plasmid for the arabinose-inducible expression of the immunity proteins Va03180 (pVa03180) or Va03170 (pVa03170), before (0h) and after (4h) co-culture with wild-type (WT) *V*. *alginolyticus* 12G01 attacker or a strain deleted for *hcp2* (Δ*hcp2*). Asterisks mark statistical significance between sample groups at t = 4h by an unpaired, two tailed student’s t-test (p<0.05).
(TIF)

S3 Fig H-NS represses VaT6SS1 activity. Expression (Cells) and secretion (Medium) of Hcp1 from *V*. *alginolyticus* 12G01 Δ*hcp2* or Δ*hcp2/*Δ*hns* derivatives containing endogenously C-terminal FLAG-tagged Hcp1 (Hcp1-FLAG) were detected by immunoblot using anti-FLAG antibodies. Cultures were grown in LB at 30°C with initial OD_600_ = 0.18. Loading control (LC) is shown for total protein lysates.
(TIF)

S4 Fig Taxonomic distribution of MIX V-effectors. Bacteria encoding MIX V-effectors. The number of strains is shown in parenthesis.
(TIF)

S1 Dataset MS results for VaT6SS2 secretome. (XLSX)

S2 Dataset MS results for VaT6SS1 secretome. (XLSX)

S3 Dataset MIX V-effectors and their genetic neighborhood among complete genomes in NCBI refseq database. (XLSX)

S1 File Detailed explanation of data presented in S3 Dataset. (DOCX)
