# Supplementary figures and images for: Type VI Secretion System Toxins Horizontally Shared between Marine Bacteria
Source: PLoS Pathog. 2015 Aug 25;11(8):e1005128. doi: 10.1371/journal.ppat.1005128 (PMC4549250; doi:10.1371/journal.ppat.1005128)

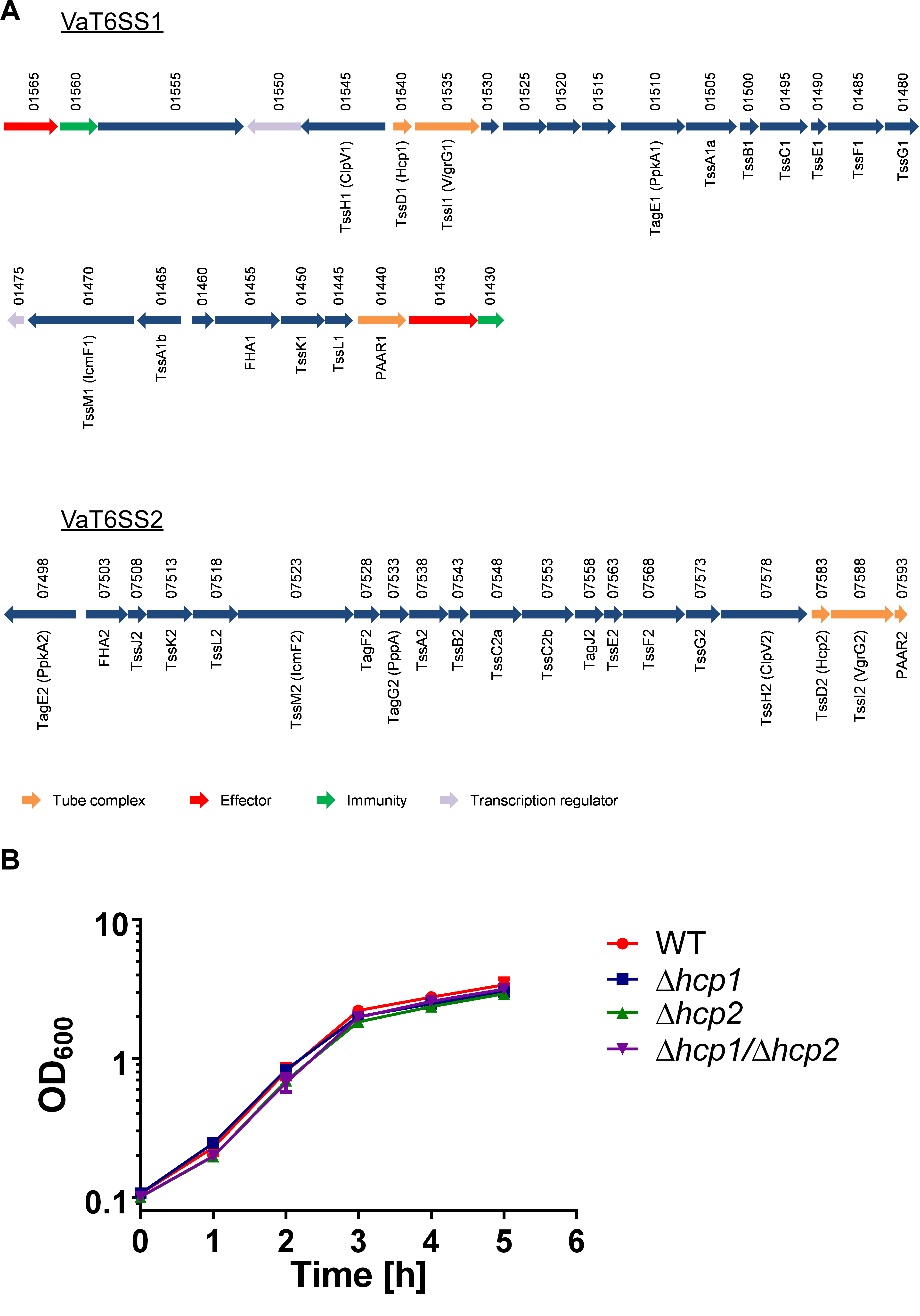

Supplement: S1 Fig — (A) Organization of the V. alginolyticus 12G01 T6SS gene clusters. Direction of gene transcription is represented by arrows. V12G01 locus numbers listed above; T6SS component name according to accepted nomenclature shown below. (B) Growth of V. alginolyticus 12G01 and derivative strains in MLB at 30°C shown as OD600 measurements. Data are mean ± SD, n = 3. WT = wild-type. (TIF) [file ppat.1005128.s001.tif]

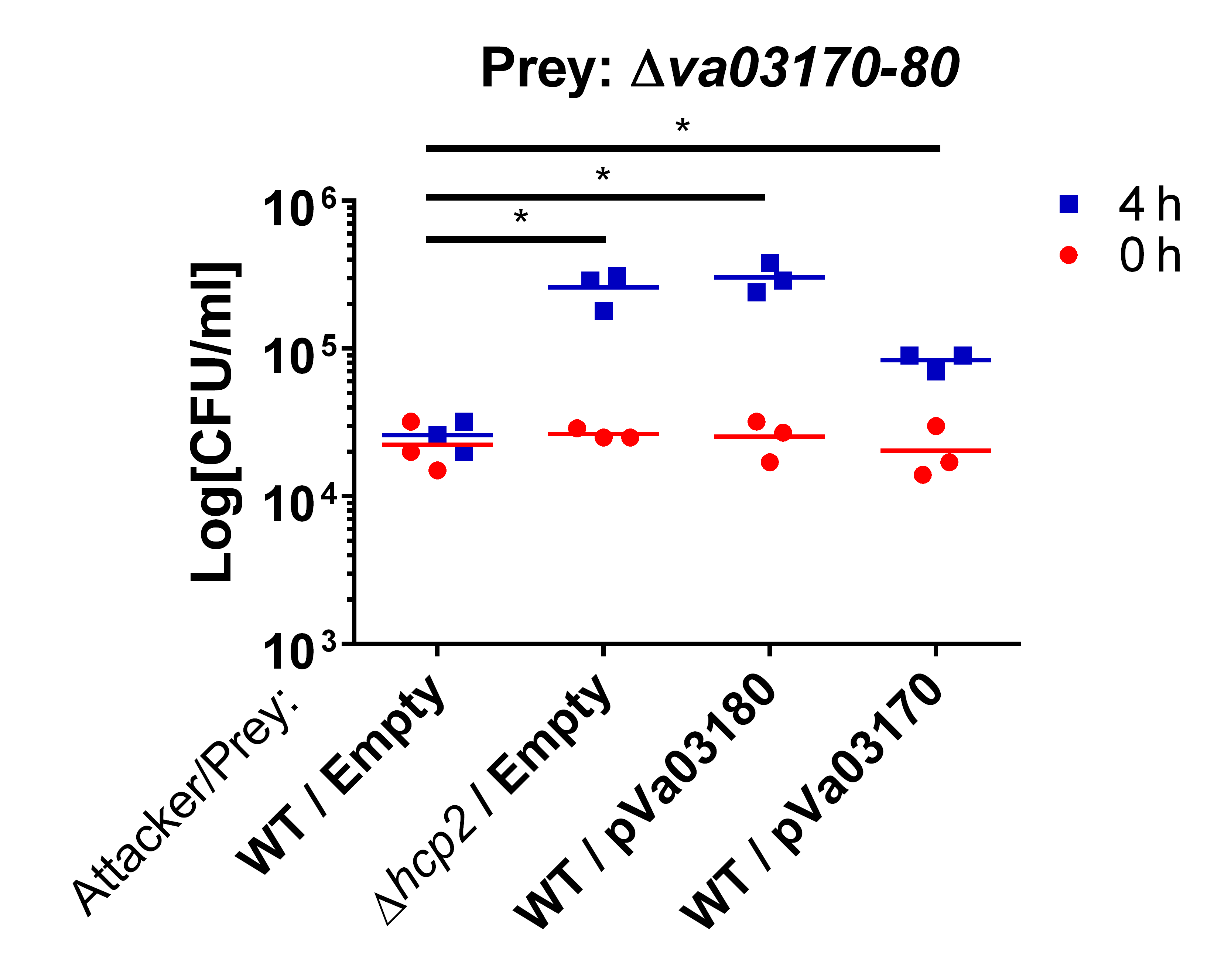

Supplement: S2 Fig — Viability counts of Δva03170-80 prey strain containing an empty plasmid or a plasmid for the arabinose-inducible expression of the immunity proteins Va03180 (pVa03180) or Va03170 (pVa03170), before (0h) and after (4h) co-culture with wild-type (WT) V. alginolyticus 12G01 attacker or a strain deleted for hcp2 (Δhcp2). Asterisks mark statistical significance between sample groups at t = 4h by an unpaired, two tailed student’s t-test (p<0.05). (TIF) [file ppat.1005128.s002.tif]

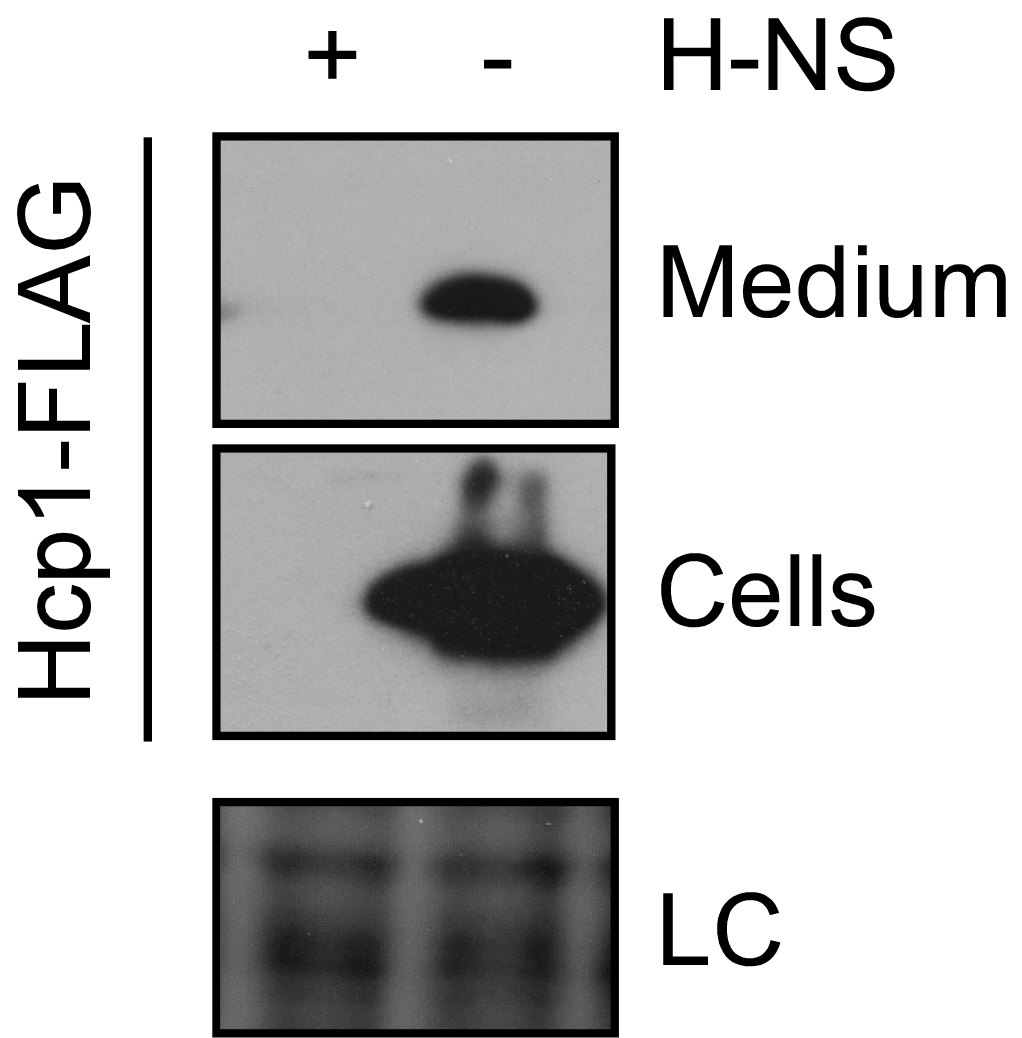

Supplement: S3 Fig — Expression (Cells) and secretion (Medium) of Hcp1 from V. alginolyticus 12G01 Δhcp2 or Δhcp2/Δhns derivatives containing endogenously C-terminal FLAG-tagged Hcp1 (Hcp1-FLAG) were detected by immunoblot using anti-FLAG antibodies. Cultures were grown in LB at 30°C with initial OD600 = 0.18. Loading control (LC) is shown for total protein lysates. (TIF) [file ppat.1005128.s003.tif]

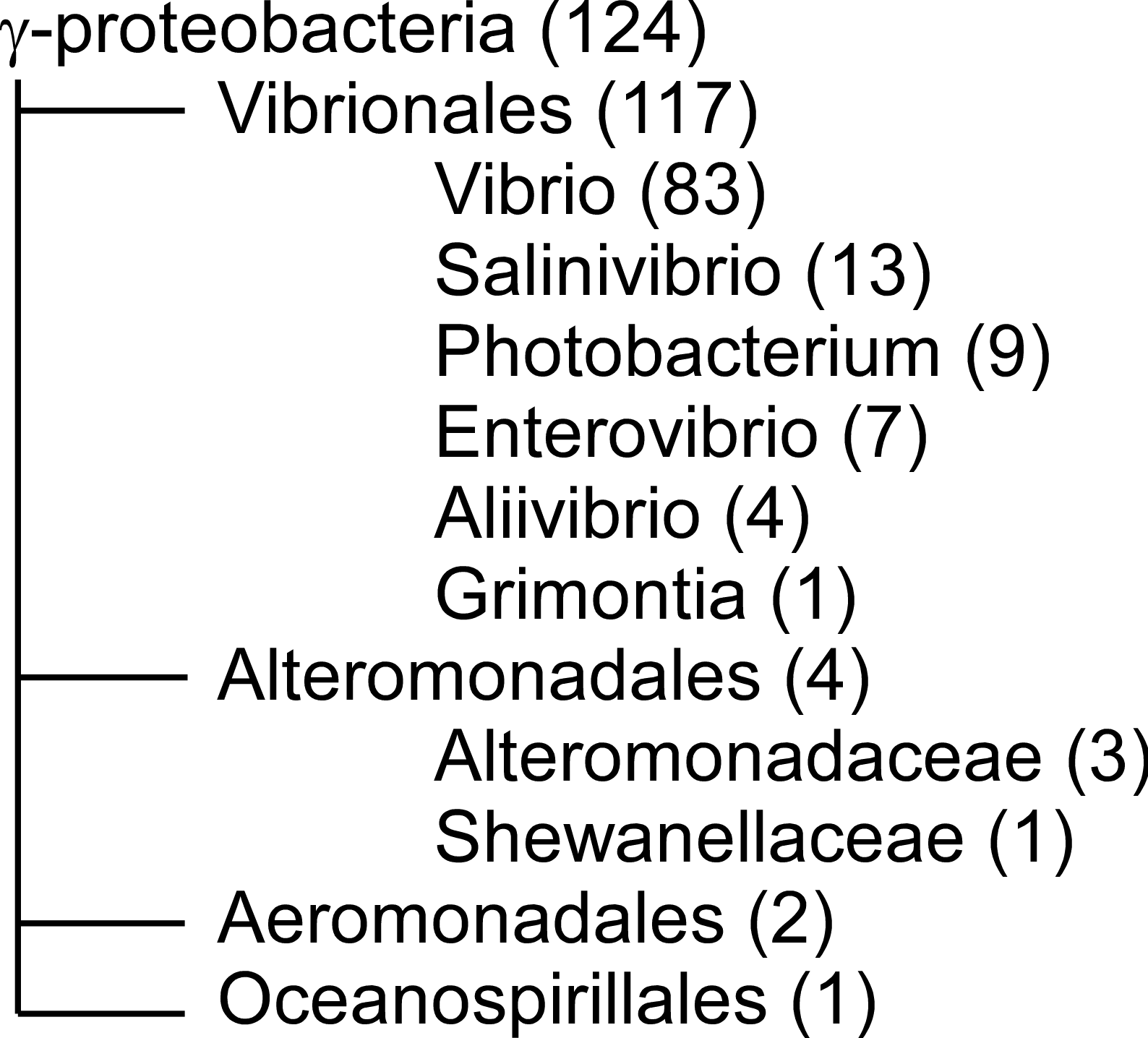

Supplement: S4 Fig — Bacteria encoding MIX V-effectors. The number of strains is shown in parenthesis. (TIF) [file ppat.1005128.s004.tif]
