# Supplementary material for: Type VI Secretion System Toxins Horizontally Shared between Marine Bacteria
Source: PLoS Pathog. 2015 Aug 25;11(8):e1005128. doi: 10.1371/journal.ppat.1005128 (PMC4549250; doi:10.1371/journal.ppat.1005128)
Supplement: S1 File — (DOCX) [file ppat.1005128.s008.docx]

**Dataset S3. MIX V-effectors and their genetic neighborhood among complete genomes in NCBI refseq database**

Sheet 1 contains MIX V genomic neighborhoods from -3 to +3 adjacent genes in the nucleotide records (labeled columns -3 to 3). An “x” denoted incomplete assembly of sequences contigs where the MIX V gene was at the end of the reads and has no complete neighborhood information. The species from which the neighborhood data was acquired is noted in the “species” column, and published T6SS components are highlighted in gray, with an x marked in the T6SS column if any neighboring genes are missing, a “y” if neighboring genes are T6SS components, and a “n” if no T6SS neighboring components were found.

Sheet 2 contains domain data from NCBI batch CDD search against the COG database. Search results include: the query GI corresponding to MIX V and neighboring protein sequence, the position specific scoring matrix ID (PSSM-ID) that corresponds to the COG domain alignments, residue ranges covering the PSSM (From) and (To), E-value, Accession, and short name of the domain hit. Some hits do not cover the entire range of the domain (Incomplete), as they are missing part of the N-terminal sequence (N), part of the C-terminal sequence (C), or both (NC).

Sheet 3 contains GI number, accession number, protein annotation, and species information for the MIX V and neighboring genes.
